# Sink or swim: Making sense of the NICE intravenous fluid guidelines as a Foundation Year Doctor

**DOI:** 10.1016/j.clinme.2026.100639

**Published:** 2026-08-30

**Authors:** Talia Patel, Calum R.A. Heslop, Milap Rajpara, Shivaali Karelia, Rakesh Patel

**Affiliations:** a University Hospitals of Derby and Burton NHS Foundation Trust, United Kingdom; b Liverpool University Hospitals NHS Foundation Trust, United Kingdom; c University Hospitals of Leicester NHS Trust, United Kingdom; d Royal Free London NHS Foundation Trust, United Kingdom; e St Mary’s University London, Barts Health NHS Trust, United Kingdom

**Keywords:** Fluid balance, Foundation Year Doctor, Guideline, Intravenous fluids, Medical education, Patient safety, Postgraduate, Undergraduate

## Abstract

Foundation Year (FY) doctors consistently report feeling underprepared when applying intravenous fluid (IVF) guidelines due to inadequate training, resources and an underemphasis of evidence-based bedside assessment techniques. Given IVFs are the most commonly prescribed substance in the NHS, especially by FY doctors, inappropriate patient assessments and written prescriptions are a patient safety threat. Whilst the NICE IVF guideline exists, it is not without nuance and there remain challenges in delivering effective IVF education and sustainable learning interventions over time. From a FY doctor perspective discussed, senior clinicians demonstrating use of guidelines more explicitly may help address the gap between guidelines and clinical practice. Ultimately, given the widespread use of IVF, there is a need for improved education to develop FY doctors’ clinical reasoning and ensure patient safety.

## Introduction

Intravenous fluids (IVFs) are one of the most commonly prescribed substances in the NHS, writing a prescription for which is a task frequently completed by Foundation Year (FY) doctors.^1^ In December 2013, the National Institute for Health and Care Excellence (NICE), published a guideline for the prescription of IVFs in adults (NICE CG174), however, subsequent audits have demonstrated poor assessment of patients’ fluid and electrolyte needs and a lack of knowledge regarding IVF prescribing; both of which have patient safety implications.^2^, ^3^, ^4^, ^5^, ^6^ Approximately 20% of patients receiving IVFs suffer serious harm, including kidney injury, pulmonary oedema and biochemical disturbance. This challenge is particularly acute in the perioperative setting with deaths directly linked to IVF mismanagement.^2^, ^3^, ^7^, ^8^

Against this backdrop, there remains an urgent need to improve IVF prescribing and management, in particular, the education and training of staff required to make fluid assessments of patients.^1^

Prescribing IVFs correctly involves prescribers applying the NICE IVFs guideline to the patient in front of them, which can be easier said than done. FY doctors often lead this aspect of patient care, requiring them to make an accurate assessment of both current and cumulative fluid status, before choosing the most appropriate type and volume of fluid to prescribe.^9^ Specific difficulties for novices such as students or FY doctors, can include recalling fluid distributions and electrolyte compositions across different compartments, as well as undertaking clinical assessments in patients presenting with multimorbidity and polypharmacy.^7^ To add to the complexity, clinical assessments may frequently be carried out in the absence of a comprehensive record of the patient’s cumulative fluid balance.^3^, ^7^

The challenge for educators is that IVFs is a complex topic requiring knowledge of physiology, pathophysiology, and pharmacology. Furthermore, novices can often forget concepts with time, meaning educators must continue to clarify misconceptions and re-teach foundational knowledge. Knowledge gaps may not be sufficiently addressed prior to graduation from medical school, resulting in newly qualified doctors entering clinical practice unprepared for the prescribing challenges described above.^3^

This paper shares our experience as both educators and learners, across a multi-professional team, discussing common areas of confusion applying the NICE IVFs guideline; identifying areas of difficulty for novice prescribers relating to bedside assessment; and effective strategies for improving IVFs education.

## The NICE guidelines and applying them to clinical practice

The NICE guidelines are a synthesis of the highest quality evidence regarding a clinical topic, and support clinicians to make evidence-based decisions regarding a patient's management. In the context of IVFs, NICE CG174 provides four algorithms to achieve effective fluid and electrolyte management, and summarises its aims through the ‘5R’s’ (Table 1):

**Table 1:** The 5 Rs of fluid prescribing.^2^ Resuscitation, routine maintenance, replacement, redistribution and reassessment form the cornerstone of safe IVF prescribing, with NICECG174 aiming to provide comprehensive guidance for achieving all five principles in a range of scenarios.

1) Resuscitation	2) Routine maintenance	3) Replacement	4) Redistribution	5) Reassessment
Restore circulating volume in hypovolaemic patients.	Meet daily water, electrolyte and glucose requirements.	Replace ongoing losses (e.g. in patients with vomiting, diarrhoea, high output stomas etc.).	Recognise and manage varying fluid distribution associated with comorbidities or acute illness.	Review clinical condition regularly and adjust IVF therapy in response.

However, the challenge with using guidelines in clinical practice is not the guideline per se, but the subjective interpretation and application of the guideline in practice at the bedside, which may vary between individuals for a given patient presentation.

Having an understanding about this reality of clinical practice, but also the differences between novice and expert prescribers, as well as knowledge of educational theory, provides a platform from which to propose some helpful teaching suggestions. Firstly, educators need to enable FY doctors to understand the purpose of guidelines and the language used within them by creating activities that involve demonstrating how to use them in clinical practice.^10^ Without this support, FY doctors may end up using guidelines as 'tramlines', adhering to the guidelines in an overly rigid way, or ‘mindlessly’ adapting their prescribing practice based upon the behaviours of others, which likely presents equal risk.^11^ Many of the questions included in guidelines are also framed as binary choices, whereas in practice there is often a degree of uncertainty, meaning individuals may need more support than the guideline offers.

Secondly, educators should ideally show FY doctors the different ways in which the same guideline may be applied in clinical practice by staff with differing levels of expertise. Many experts may not ostensibly make use of a guideline for a variety of reasons.^12^ For example, in the case of a complex or atypical presentation, someone with clinical expertise may judge the guideline to have limited value. Likewise, experts may also not use an ABCDE approach to assess whether a patient is hypovolaemic and needs fluid resuscitation, arriving at the diagnosis despite none of the indicators above being obvious or apparent to a novice.^5^, ^13^ In these situations, novices could perceive experts as ignoring the guideline without sufficient explanation of clinical reasoning by experts. Therefore, experts should explain their management strategy to novices, highlighting the differences and reasoning behind their approach when this happens.^14^

Thirdly, IVF prescribing requires knowledge about electrolytes, which can vary in complexity with different scenarios. Sound understanding of electrolyte physiology is necessary as fluid composition varies across different types of fluid. Furthermore, various clinical presentations, such as diabetic ketoacidosis, require particular prescriptions to be adjusted according to the evolving situation.^9^ As well as understanding when to monitor electrolyte balance, prescribers must also know how to respond should they be imbalanced. Furthermore, IVF prescribing, rather than being one discrete task, usually involves multiple evolving tasks including assessment and reassessment, which junior prescribers may struggle to manage alongside other workplace pressures.^13^

Finally, educators need to ensure that FY doctors gain experience prescribing in different specialties, as experience prescribing in one clinical subspecialty may be insufficient for safely prescribing in another. For example, the fluid prescription for an otherwise young and well patient on a general surgical ward is likely to be very different to that of an elderly patient on a cardiology ward, especially if the patient has established cardiovascular disease, such as heart failure. This is a significant challenge faced by FY doctors in particular, who lack experience and have multiple learning commitments.

## Bedside clinical assessment

Effective use of the NICE IVFs guideline requires a thorough bedside clinical assessment including history-taking, physical examination and laboratory investigations review. The first assessment described by the guideline is to use an ABCDE approach.^2^ Focused history-alongside can also be used for assessing either fluid or electrolyte needs (i.e. previous limited intake, thirst, abnormal losses, comorbidities). Whilst experts may understand how these indicators influence IVFs prescription, novices may not. For example, students or FY doctors on call, may be unfamiliar with what ‘normal’ vs ‘abnormal’ losses may be present, having not regularly looked after, or provided continuity of care for any period of time to the patients. The term ‘comorbidities’ can also be ambiguous, as conditions are not equally relevant when prescribing IVFs. For example, patients with comorbidities such as eczema or glaucoma require fewer extra considerations compared with patients with established kidney disease requiring dialysis or liver cirrhosis with ascites.

At the end of any history-taking and clinical examination process, consideration then needs to be given to the way in which patient-reported signs and symptoms are translated into medical jargon. In the context of IVF prescription, there are numerous terms used when communicating fluid status such as ‘under-filled’, ‘overloaded’, ‘wet’, ‘dry’, ‘positive balance’ and ‘negative balance’. The above terms are jargon relying on both knowledge and understanding about the assessment of fluid status in clinical practice. Judgements can be subjective, sometimes with minimal history taking and unreliable clinical signs used to evaluate fluid status. An important part of any future IVFs teaching may therefore include opportunities for FY doctors to share their assessments and receive targeted feedback about their presentation by more experienced clinicians.

With regards to bedside examination, the NICE IVFs guideline presents a number of indicators for assessing volume status: systolic blood pressure <100 mmHg, heart rate >90 beats per minute; capillary refill >2 s; respiratory rate >20 breaths per minute; National Early Warning Score ≥5; and 45° passive leg raising suggests fluid responsiveness.^2^ There are also several clinical examinations not included in guideline such as skin turgor, jugular venous pressure, and mucous membrane assessment. However, evidence from the wider literature would suggest the sensitivity of many indicators for hypovolaemia included in the guideline and traditional clinical examination manoeuvres is poor.^15^ Whilst a moist tongue may reduce the likelihood of prescribing IVFs following evaluation, appearances alone are not a sensitive or specific sign for dehydration.^16^, ^17^

Far more useful, is when symptoms such as dizziness on standing for more than a minute are combined with clinical signs such as a drop in blood pressure over the same period and a rise in pulse rate of more than 30%.^6^, ^15^ Through the lens of evidence-based clinical examination, very few of the indicators in the guideline for assessing volume status are sufficiently sensitive or specific to confirm a diagnosis of hypovolaemia. The clinical assessment of volume status in patients with multimorbidity can also be challenging in practice. The physical condition of patients may mean staff are unable to manoeuvre patients, especially supporting them to get in the correct position or lifting limbs whilst fluid boluses are being administered. Often there are circumstantial challenges such as patients being placed on trollies in corridors or at the back of an ambulance which can also make these manoeuvres less feasible to complete.

Laboratory investigations can also be a helpful in the assessment of fluid balance. For example, NICE IVFs guideline states an increase in urea or creatinine by more than 50% in the absence of other causes as a reliable indicator of hypovolaemia and acute kidney injury.^2^ That said, biochemical markers shouldn’t be interpreted in isolation and should be clinically correlated with other examination findings (Table 2).^2^, ^18^

**Table 2:** Fluid status assessment table; a synthesis of evidence-based parameters as described in The Journal of the American Medical Association^17^, ^19^ *^1^ Both pulse increment >30 beats per minute and postural hypotension are significant findings in those with suspected blood loss. *^2^ Presence of a dry axilla is particularly indicative of hypovolaemia in elderly patients. *^3^ Absence of dry mucous membranes, furrows in the tongue and sunken eyes indicate hypovolaemia is less likely. *^4^ Point of care ultrasound findings alongside chest radiography are also helpful in identifying overload, but there is reduced routine utility in FY doctors. Evidence base and utility can vary with each parameter as detailed above in different populations (e.g. elderly patients vs pregnant patients). Findings should therefore be interpreted holistically alongside biochemistry to guide judgments regarding fluid status.^2^, ^17^, ^19^

Assessment domain	Hypovolaemia	Hypervolaemia
Clinical Examination Findings	Pulse increase increment >30 BPM *^1^	Crackles on auscultation
	Postural hypotension >20 mmHg drop in systolic blood pressure *^1^	Lower extremity oedema
	Dry axilla *^2^	JVP >3 cm above sternal angle
	Dry mucous membranes of mouth and nose*^3^	
	Furrows in the tongue*^3^	
	Sunken eyes*^3^	
	Upper or lower extremity weakness	
	Non-fluent speech	
	Confusion present	
Laboratory Findings	Raised serum urea levels (mmol/L)	B-Type Natriuretic Peptide >100 ng/mL
	Raised creatinine levels (µmol/L)	
Radiographic findings		Pulmonary congestion on chest radiographs: Interstitial oedema and/or peribronchial cuffing *^4^

If no additional fluid needs are identified, routine maintenance fluids can be prescribed as per algorithm three.^2^ Alternatively, if there are abnormal losses or redistribution then users can follow algorithm four to tailor fluid regimes appropriately^2^, ^5^

Finally, the NICE IVFs guideline requirement for a robust reassessment of fluid status when ongoing IVFs are given >24 h, is the final aspect of good fluid management. Re-examination and re-appraisal are critical safety-netting behaviours as well, since reviewing physiological response and fluid balance can also help inform whether to continue with IVF therapy or appropriately discontinue it.

## Foundation doctor perspective

As a FY doctor rotating through paediatrics, general surgery and healthcare of older people, I have had the opportunity to observe fluid prescribing practices across a wide range of patient ages spanning from neonates to patients in their nineties. In paediatrics, fluid status and prescribing are, understandably, closely monitored and tightly controlled. Signs of dehydration – present or absent – are routinely documented, and fluid prescriptions are calculated based on weight (Holliday-Segar formula) meaning careful consideration is given prior to a fluid administration.^20^ Acute kidney injury (AKI) is uncommon among children and actively prevented against by carefully prescribing using the age-appropriate IVF guideline.

In contrast, within adult medicine there is only one IVF guideline, and whilst significant co-morbidities and age are discussed, in practice, neither the NICE guideline, nor trust guidelines are consistently used and documented prior to IVF prescription. The paucity of comprehensive fluid balance assessments is demonstrated in audit data; only 26% of patients received a documented fluid balance assessment prior to prescription of routine maintenance fluids in an audit of a large local NHS trust.^5^ Furthermore, there were no fluid balance monitoring plans in the majority of patients.^5^ Lack of detailed assessment and management plans, alongside incomplete fluid charts can pose significant barriers to safe prescribing and reflect how fluid status is not always a priority within patient care.

This experience may be unique, or atypical, however resonate with general finding that FY doctors feel underprepared for prescribing fluids, especially when the reality of clinical practice doesn’t necessarily align with structured teaching before graduation, or simple patient scenarios that don’t reflect day-to-day challenges.^3^ Given IVFs are the most commonly prescribed drug in the NHS, greater emphasis could be placed on making the clinical reasoning underpinning IVF prescribing more visible, as well as creating a culture in which the NICE IVFs guideline is used more explicitly in clinical practice.^21^

## Educational interventions

Evidence investigating the effectiveness of IVF education is mainly represented by interventions for medical prescribers and targeting increase in fluid and electrolyte knowledge.^13^ Likewise, interventions do not address the contextual factors individuals face when prescribing in practice, such as communication, workplace demands and poor documentation.^8^, ^13^ Multiple interventions aimed at resident doctor workflow in the NHS are described in the existing literature; for example, improvement in IVF prescribing has been achieved through introducing prescription aid lanyards, specialised IVF prescription charts, and daily review template sheets in various trusts.^5^, ^6^, ^21^ By comparison, there is a paucity of evidence investigating the effectiveness of IVF education in medical students.^6^, ^21^ Whilst precision teaching methods such as SAFMEDS (Say *A*ll *F*ast *M*inute *E*very *D*ay *S*huffled) demonstrated an increase in knowledge recall about IVFs, the impact on perceived and actual competence in practice is unknown^21^ and does not necessarily address practical gaps in confidence prescribing.^6^ Given many undergraduate courses are at least four years in length, a spiral curriculum approach to IVF education may be beneficial where early years teaching on physiology and pharmacology could be supplemented with more fluid balance challenges from clinical practice, and where later years teaching in paediatrics, surgery and care of the older person could be optimised by challenging students to recall basic science first principles to emphasise what safe IVF prescribing looks like in reality.^6^, ^21^ IVF education does take place in Foundation Training and could be optimised by providing more insights from experts around difficult prescribing challenges across different clinical contexts. There is now an opportunity to improve the quality of research in this area. The heterogeneity of educational interventions can make them difficult to compare, and often outcome measures are limited to pre- and post-intervention study designs or qualitative feedback from learners.^6^ Longer term analysis through clinical audit, quality improvement and research across both undergraduate and postgraduate medical education, are required to measure knowledge and skill retention as a result of these interventions, and to determine the effectiveness of educational interventions.^3^, ^4^, ^21^

## Conclusion

The purpose of this piece was to highlight some of the challenges to implementing the NICE IVFs guideline in practice through the lens of education focusing on areas where FY doctors may struggle. There is a need to re-examine the way we teach bedside clinical skills such as history-taking and physical examination by making visible links between the background science and clinical context to develop clinical reasoning. Finally, there remain too few education and training interventions described that demonstrate improved learning outcomes over time. Poor knowledge recall and skills decay is a real problem, and better interventions are required to ensure avoidable harm is minimised and patient safety overall is improved, as well as further study to identify the most successful combinations of education interventions.

## CRediT authorship contribution statement

**Talia Patel:** Writing – review & editing, Writing – original draft, Project administration, Conceptualization. **Calum R.A. Heslop:** Writing – review & editing, Writing – original draft. **Milap Rajpara:** Writing – review & editing. **Shivaali Karelia:** Writing – review & editing. **Rakesh Patel:** Writing – review & editing, Writing – original draft, Supervision, Conceptualization.

## Funding

No funding was received for the preparation of this article.

## Declaration of competing interest

The authors declare that they have no known competing financial interests or personal relationships that could have appeared to influence the work reported in this paper.
